# Health education interventions to raise awareness of rheumatic fever: a systematic review protocol

**DOI:** 10.1186/2046-4053-2-58

**Published:** 2013-07-18

**Authors:** Laura Susan Ramsey, Lauren Watkins, Mark Emmanuel Engel

**Affiliations:** 1 Department of Medicine, Faculty of Health Sciences, University of Cape Town, Anzio Road, Observatory 7925, Cape Town, South Africa

**Keywords:** Acute rheumatic fever, Awareness, Health education, Pharyngitis, Rheumatic heart disease

## Abstract

**Background:**

There is a significant global health burden associated with acute rheumatic fever (ARF) and rheumatic heart disease (RHD), especially in developing countries. ARF and RHD most often strike children and young adults living in impoverished settings, where unhygienic conditions and lack of awareness and knowledge of streptococcal infection progression are common. Secondary prophylactic measures have been recommended in the past, but primary prevention measures have been gaining more attention from researchers frustrated by the perpetual prevalence of ARF and RHD in developing countries. Health education aims to empower people to take responsibility for their own well-being by gaining control over the underlying factors that influence health. We therefore conducted a review of the current best evidence for the use of health education interventions to increase awareness and knowledge of streptococcal pharyngitis and ARF.

**Methods and design:**

This article describes the protocol for a systematic review of the effectiveness of health education interventions aimed at increasing awareness and knowledge of the symptoms, causes and consequences of streptococcal pharyngitis, rheumatic fever and/or rheumatic heart disease. Studies will be selected in which the effect of an intervention is compared with either a pre-intervention or a control, targeting all possible audience types. Primary and secondary outcomes of interest are pre-specified. Randomized controlled trials, quasi-randomized trials, controlled before–after studies and controlled clinical trials will be considered. We will search several bibliographic databases (for example, PubMed, EMBASE, World Health Organization Library databases, Google Scholar) and search sources for gray literature. We will meta-analyze included studies. We will conduct subgroup analyses according to intervention subtypes: printed versus audiovisual and mass media versus training workshops.

**Discussion:**

This review will provide evidence for the effectiveness of educational components in health promotion interventions in raising public awareness in regard to the symptoms, causes and consequences of streptococcal pharyngitis, ARF and/or RHD. Our results may provide guidance in the development of future intervention studies and programs.

## Background

Acute rheumatic fever (ARF) is a consequence of inadequate treatment of skin or pharyngeal infection with group A streptococcus (GAS) [1]. ARF requires secondary prophylaxis of penicillin to prevent recurring episodes, as well as to prevent the possibility of developing the most serious consequence of rheumatic heart disease (RHD) [2]. It is estimated that at least 15 million people are currently living with RHD. Each year approximately 282,000 new cases develop and 233, 000 deaths may be attributed to the disease [3]. ARF and RHD most often strike children and young adults living in poverty, where overcrowding, undernutrition and lack of proper healthcare and awareness of the disease are common. Approximately 0.5 per 1,000 children living in developed countries have RHD, whereas in sub-Saharan Africa as many as 5 to 7 per 1,000 children are affected [4].

The World Health Organization (WHO) guidelines recommend secondary prophylaxis as the most effective method of reducing the burden of ARF and RHD [1]. Although recent studies have shown that, by the time susceptible patients receive clinical attention for secondary prophylactic measures, they often have already accumulated significant valve damage from unrecognized attacks of ARF [5].

Primary prevention measures have been gaining more attention from researchers frustrated by the perpetual prevalence of ARF and RHD in developing countries around the globe [6]. Recent investigations have found that primary prevention is actually more successful and cost-effective [7]. Primary prevention involves a single injection of benzathine penicillin after confirmation of streptococcal pharyngitis by microbiological culture of a throat swab specimen. Antibiotic treatment of GAS infection has been shown to reduce the attack of ARF by 70%, with intramuscular penicillin reducing it by as much as 80% [5].

Research has shown that when knowledge of the disease among a population increases, the number of reported cases increases and thus the number of patients treated increases [8]. By following The Ottawa Charter for Health Promotion, established in 1986, health advocates have made significant strides in treating afflicted populations around the world [9]. Several strategies promoting the importance of seeking primary prevention treatment (that is, presenting at a clinic or doctor’s room for a throat swab evaluation) of streptococcal pharyngitis through education have been reported [10,11]. These include multifaceted programs incorporating pamphlets, posters, videos, television and radio advertisements, heart models and training workshops. Previous interventions have targeted various types of participants, including schoolchildren, parents, healthcare workers, teachers, community members and patients. These interventions or multifaceted combinations of interventions may need to be utilized to promote ARF and RHD awareness and prevention.

Despite the many studies reporting success in health education interventions to promote awareness of pharyngitis and ARF, a high prevalence of ARF and RHD persists in many populations [3]. We therefore propose to conduct a review of the current best evidence for education interventions to promote awareness of streptococcal pharyngitis and rheumatic fever.

## Methods and design

The review protocol has not been registered in any prospective register of systematic reviews.

### Criteria for considering studies for this review

#### *Types of studies*

We will include studies that compare the effects of an intervention with either the pre-intervention findings or a control. Studies must have been published after 1986, in accordance with the declaration of the Ottawa Charter.

#### *Types of participants*

Participants will be schoolchildren, patients, educators or healthcare workers in school, community, city and nationwide settings.

#### *Types of interventions*

Interventions must include as an objective an educational aspect of streptococcal pharyngitis, ARF or RHD that contains information relating to symptoms, causes and consequences. We will endeavor to extract the health education component in instances where interventions are presented as multifaceted interventions, for example, multimedia or active surveillance. Should this not be possible, such interventions will be presented in a narrative fashion.

#### *Types of outcome measures*

Results must include quantitative data for outcomes measured.

### Primary outcomes

The primary outcomes are as follows: increase in throat-swabbing rates, increase in awareness of ARF and increase in knowledge of symptoms and consequences of streptococcal pharyngitis, ARF and RHD.

### Secondary outcomes

The secondary outcomes are increase in the number of ARF cases reported to hospital or health facilities, decrease in the incidence and severity of ARF and RHD, reduction in the number of recurrent attacks of ARF and success in regular compliance with secondary prophylaxis.

### Search methods for identification of studies

Extensive searches will be performed by LR and LS with the help of ME to collect all relevant studies available by May 2013. We will include both peer-reviewed journal articles and the gray literature in our searches. Only the English-language literature will be included in the searches.

### Electronic searches

We will search the following electronic databases: PubMed, EMBASE, WHO Library databases and Google Scholar. We will use both text words and medical subject heading (MeSH) terms, for example, rheumatic fever, rheumatic heart disease, streptococcus pyogenes, GAS, group A strep*, streptococ*, pharyngitis, sore throat, awareness, health promotion, intervention and health educat*. These terms will be used in varying combinations. Table 1 shows the main search strategy we will use.

**Table 1:** **PubMed search strategy, modified as needed for use in other databases**^a^

**Search**	**PubMed**
1	(awareness) OR (“health promotion”) OR (intervention) OR (“health educat*”)
2	(“rheumatic fever” [MeSH]) OR (“rheumatic heart disease” [MeSH]) OR ([tiab])
3	(streptococ*) OR (“group A strept*”) OR (GAS) OR (“strept* pyogenes”)
4	(“sore throat”) OR (pharyngitis) OR (“throat infection”)
5	1 AND 2
6	1 AND 3
7	1 AND 4
8	5 AND 6 AND 7

*, wildcard term as per database notation; ^a^MeSH, medical subject heading.

### Conference proceedings

We will search the following conference proceedings for relevant abstracts: International Union for Health Promotion and Education, the World Congress of Cardiology, and the Pan African Society of Cardiology.

### Manual searches

We will obtain the reference lists of the selected relevant studies. The full-text articles analyzed for inclusion in the review will be evaluated for further information.

### Data collection and analysis

The methods for data collection and analysis will be based on the guidelines of the *Cochrane Handbook of Systematic Reviews for Interventions*[12].

### Selection of studies

We will construct a screening guide to make certain that the inclusion criteria are followed and consistently applied by all review authors. Two review authors (LR and LS) will work individually to screen the titles and abstracts to determine the eligibility of all studies identified through the literature searches. LR will obtain the full text of studies considered potentially eligible. LR and LS will independently review the full text using inclusion criteria to evaluate eligibility before comparing results. Any discrepancies in inclusion or exclusion will be resolved through discussion or through consultation with a third author (ME). Reasons for exclusion will be documented and reported.

### Data extraction and management

References will be managed using Thomson ISI ResearchSoft EndNote X2 software (Thomson Reuters, New York, NY, USA). Two authors will independently extract data from each included article using a standardized data collection form, resolving any incongruities by discussion and agreement. If no consensus can be reached, a third author (ME) will intercede. LR will enter the final data into Cochrane Collaboration Review Manager (RevMan) version 5.1 statistical software (http://ims.cochrane.org/RevMan). ME will cross-check the entered data to confirm accuracy.

### Assessment of risk of bias in included studies

Each included study will be assessed for risk of bias. Two authors will independently assess the methodological quality of the studies in accordance with the methods used by the Cochrane Collaboration [12] and the Cochrane Consumers and Communication Review Group [13]. We will appraise *inter alia* the study design, sampling strategy, method of determining outcomes and appropriateness of the analytical methods used.

The criteria used to assess the risk of bias for randomized controlled trials will include the following elements: random sequence generation, allocation sequence concealment, blinding (outcome assessment), incomplete outcome data, selective outcome reporting and other sources of bias. Blinding criteria will not be included in the risk of bias assessment, because blinding of participants and investigators is not feasible in a health education intervention. If quasi–randomized controlled trials, controlled before and after studies or interrupted time series studies are included in the review, we will assess their risk of bias accordingly, utilizing adaptations to the above criteria by incorporating suggestions made by the Cochrane Consumer and Communication Review Group [13]. Any disagreements will be resolved by discussion and consensus in consultation with the third author to resolve persistent inconsistencies.

### Measures of treatment effect

Data analysis will be completed using Cochrane Collaboration Review Manager version 5.1 statistical software. For dichotomous data, we will calculate risk ratios and their corresponding 95% confidence intervals and *P* values. For CBA studies, we will report relative post-intervention percentage changes and standardized mean differences. We anticipate that data points will primarily be pre- and post-intervention. Where follow-up data were collected at further follow-up, we will report results taken from the furthest points in time relative to the intervention.

### Dealing with missing data

In the cases of absent or incomplete evidence found in the included studies, study authors will be contacted for further information.

### Data synthesis and assessment and investigation of heterogeneity

Heterogeneity between trials will be assessed using the χ^2^ test set at a 10% level of significance. The impact of any statistical heterogeneity will be quantified using the *I*^2^ statistic [12]. If there is an acceptable degree of heterogeneity and it is appropriate to pool the data, the Mantel-Haenszel statistical method and random effects analysis model will be used for analysis. The results will be presented in the form of a meta-analysis. If we are unable to combine the studies due to varying study designs or to heterogeneity in selection of participants, coexisting interventions, attrition or detection methods, the data will be presented in narrative form. We will perform subgroup analyses by intervention subtypes: printed versus audiovisual and mass media versus training workshops.

### Sensitivity analyses

Sensitivity analyses will be performed to determine whether the study design could influence the results of the meta-analysis and to determine the impact of excluding studies with a high-risk bias on the results.

### Presenting and reporting of results

Information obtained from this systematic review will be presented in various ways. The study selection process will be summarized by means of a flow diagram. The κ statistic will be used to assess agreement between the full-text screening, data extraction and risk of bias assessment by two authors (LR and ME). Summary tables will detail findings and risk-of-bias tables. Forest plots will also be used where appropriate. Narrative reporting will be used where outcomes lack quantitative data. Excluded studies with reasons for exclusion will also be provided.

## Discussion

### Expected significance of the study

A number of authors have reported that health education interventions are effective at increasing awareness of disease control and promoting healthcare-seeking behavior in populations with ARF and RHD [8,10,14]. Despite this, there is an incredible lack of knowledge of ARF symptoms and treatment in developing countries, and thus a high prevalence of ARF and RHD persists [4]. The findings of this systematic review will have implications for policy, practice and research. Our results will provide evidence of whether educational interventions have a significant effect on raising public awareness and knowledge regarding symptoms, causes and consequences of streptococcal pharyngitis and ARF. In addition, we intend to identify ideas or specific aspects of multifaceted interventions that should be taken into consideration in future studies, such as the most effective educational strategy or target population.

## Abbreviations

ARF: Acute rheumatic fever; CBA: Controlled before and after study; GAS: Group A streptococcus; MeSH: Medical subject heading; RHD: Rheumatic heart disease; WHO: World Health Organization.

## Competing interests

The authors declare that they have no competing interests.

## Authors’ contributions

ME conceived of the review. All authors developed the design of the protocol and will be involved in data acquisition. LR undertook the drafting of the manuscript. LR and ME will analyze the data and participate in the interpretation of the results. All authors have given their approval for publication.

## Authors’ information

LR, BSc (Biology), Global Health Certificate, is a visiting research fellow. LS holds a BA degree in English Literary Studies and Media & Writing. ME, MPH (Epidemiology), PhD (Medicine), is a senior researcher focusing on RHD.
